# Controlling Plasma-Functionalized Fillers for Enhanced Properties of PLA/ZnO Biocomposites: Effects of Excess l-Lactic Acid and Biomedical Implications

**DOI:** 10.1021/acsami.4c20196

**Published:** 2025-03-12

**Authors:** Daniel A. L. V. Cunha, Felippe M. Marega, Leonardo A. Pinto, Eduardo H. Backes, Teresa T. Steffen, Larissa A. Klok, Peter Hammer, Luiz A. Pessan, Daniela Becker, Lidiane C. Costa

**Affiliations:** † Graduate Program in Materials Science and Engineering, Federal University of Sao Carlos, Sao Carlos, 13565-905, Brazil; ‡ Department of Materials Engineering, Federal University of Sao Carlos, Sao Carlos, Sao Paulo 13565-905, Brazil; § Institute of Chemistry, Sao Paulo State University, Araraquara 14800-900, Brazil; ∥ Graduate Program in Materials Science and Engineering, State University of Santa Catarina (UDESC), Joinville 88.035-901, Brazil; ⊥ Center for Characterization and Development of Materials, Federal University of Sao Carlos, Sao Paulo 13565-905, Brazil

**Keywords:** poly(lactic acid), zinc oxide, biocomposites, plasma surface treatment, excess functionalizing agent, washing, material properties

## Abstract

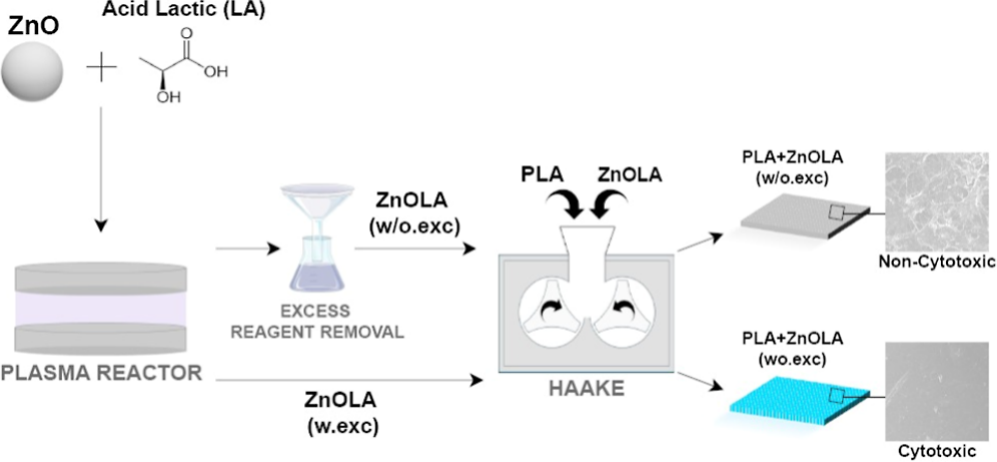

Plasma surface treatment of ceramic particles has emerged as a promising approach for developing biocomposites intended for use in tissue engineering applications. Introducing functional groups on particle surfaces promotes changes in material surface properties, enhancing adhesion, biocompatibility, and reactivity. It can also mitigate degradation during the processing of polymer matrices in composite materials. Therefore, carefully choosing the functionalizing agent responsible for generating the functional groups and selecting appropriate functionalization parameters are significant steps in the plasma surface treatment process. However, in a tissue engineering context, an excess of the functionalizing agent can be harmful, increasing cell toxicity and inhibiting the stimulation of cell growth, consequently delaying or even hindering tissue regeneration. This article examines how the functionalizing agent excess of l-lactic acid (LA) applied in the plasma surface treatment of the filler affects the thermal, rheological, biological, and wettability properties of poly(lactic acid) (PLA) and zinc oxide (ZnO) biocomposites. The investigation reveals that the surface treatment effectively mitigated the catalytic effects of ZnO on PLA degradation during melt processing, regardless of the excess functionalizing agent. There was minimal impact on the material’s rheological, thermal, and wettability characteristics, but the LA residue significantly influenced cell proliferation and the biological response. These findings show the importance of removing excess functionalizing agents to obtain biocomposites suitable for tissue engineering applications.

## Introduction

Biomaterials are of considerable interest and play a key role in various biomedical applications, including regenerative medicine and tissue engineering (TE).^1^ Within this field of research, living cells are grown in specific structures to create functional substitutes for damaged tissues or organs.^1,2^ Substantial advancements have been achieved in TE and contemporary medical practices, focusing on biomaterials tailored to integrate seamlessly with the body.^3^ Ensuring that the properties of implanted biomaterials remain stable during the treatment period is fundamental to guaranteeing continued function and tissue regeneration.^4^ In this context, the challenge is to develop functional biomaterials with adequate biological properties and a favorable structure capable of stimulating cell adhesion, growth, and the formation of new tissues.^5^

Biocomposites consisting of a biodegradable polymer matrix and bioceramics have proven effective in various biomedical applications.^6^ The biocompatibility and osteoconductivity of bioceramics, combined with the relatively high mechanical properties of polymers, make them ideal for the development of implantable structures.^7^ Therefore, poly(lactic acid) and zinc oxide (PLA/ZnO) biocomposites are highly promising formulations. PLA is an ideal polymer matrix for medical devices and diverse other applications due to its excellent processability and unique properties, such as biocompatibility and biosorption.^3,8^ Furthermore, it is the most widely used biodegradable polymer, offering superior mechanical strength and elastic modulus compared to other biodegradable polymers, and it is generally recognized as safe (GRAS) by the FDA.^9^ Its biodegradation behavior is a critical attribute that underscores its suitability for medical and industrial applications, making it particularly attractive for the development of sustainable biomedical materials.^9,10^ Incorporating ZnO fillers further enhances the properties of the polymer matrix, giving it antibacterial properties and bioactivity capable of promoting the regeneration process of various types of cells and tissues.^11,12^ Due to these advantages, PLA/ZnO biocomposites have been targeted for different applications, especially in the biomedical field. Studies highlight their effectiveness in wound healing, bone regeneration, drug release, and implant coatings.^12^ For instance, Radwan-Pragłowska et al. demonstrated that hybrid nanofibrous scaffolds, consisting of PLA doped with ZnO nanoparticles and acylated chitosan, support wound healing by ensuring adequate water vapor permeability and biodegradability, key factors for skin tissue engineering. The study also revealed that the improved conductivity imparted by ZnO enhanced cell proliferation during electrical stimulation.^13,14^ Harb et al. investigated PLA-based scaffolds with ZnO and tricalcium phosphate (TCP), finding that the addition of ZnO significantly increased surface roughness, which in turn enhanced protein adsorption by up to 85%. This improvement facilitated mesenchymal stem cell proliferation and promoted osteogenic differentiation, as evidenced by elevated alkaline phosphatase activity.^15^ These findings underscore the versatility and potential of PLA/ZnO biocomposites in diverse biomedical fields.

While PLA/ZnO biocomposites offer significant advantages, certain challenges still hinder their widespread application. For example, in TE, achieving an optimized ratio between the inorganic filler and the polymer matrix and its availability is essential for bone formation and regeneration, as its contact with cells and fluids can be hindered, and the degradation rate must be tailored with tissue restoration and healing.^15,16^ Therefore, material stability during processing and when implanted, is a critical factor in controlling the degradation of PLA/ZnO biocomposites.^17^ Recent studies have reported significant degradation of the PLA polymer matrix, particularly when in the molten state, driven by multiple mechanisms catalyzed by ZnO. Qu et al. demonstrated that incorporating ZnO nanoparticles can catalyze the hydrolytic degradation of PLA even at temperatures below its glass transition temperature (*T*_g_). According to the authors, the activation energy for the hydrolysis of PLA favored by ZnO is around 38% lower than the hydrolysis of neat PLA.^18^ Hydrolytic degradation of PLA is facilitated by the carboxyl end groups generated after the cleavage of the ester bond, leading to the formation of acidic products that can further accelerate the deterioration of the polymer.^19^ Simultaneously with hydrolytic degradation, PLA degradation occurs through the release of Zn^2+^ ions, which reduce the activation energy of the process, especially under conditions of high shear rates and temperature.^20^ Lizundia et al. propose that, under these conditions, intermolecular depolymerization and transesterification reactions are triggered, resulting in a severe reduction in the molar mass, thermal stability, and physical properties of PLA.^21^ Similar results were already observed by our group when melt compounding PLA with Biosilicate, which also, during processing, can release significant cations (Ca^2+^) and results in considerable thermoregulation.^17^

One promising strategy to reduce the autocatalytic degradation of PLA in biocomposites with ZnO is to modify the surface of the particles. This approach, particularly when using plasma surface treatment, has emerged as an effective alternative for biomedical applications as it avoids the need for potentially toxic solvents.^19^ Plasma surface is a versatile treatment that allows the introduction of specific groups, such as carbonyls, amines, and hydroxyls, onto the surface of the particles.^22,23^ This provides a protective effect that can control the release of degradation catalyst ions, thereby increasing the stability of the fillers and their interaction with the polymer.^17^ The incorporation of functional groups generally occurs through the dynamic balance between the material’s surface conditioning and active species in the plasma activation medium. When these activated species reach the surface of the substrate, the breaking of the molecular chain can result in the formation of new functional groups. Therefore, the recombination of atoms to activate the surface depends on the functionalizing agent, the selection of which must consider the desired properties of the particles and the process conditions.^24,25^

Incorporating carboxylic acid functional groups, derived from l-lactic acid and acrylic acid, for example, is an attractive alternative for biomedical applications due to their known ability to promote cell adhesion, proliferation, and differentiation.^25^ Previous studies have revealed that acrylic acid plasma-derived polymers can sustain high levels of cell attachment, both of cell lines and primary cells.^26^ Furthermore, research highlights the potential of these functional groups to reduce degradation effects in PLA polymer matrices when combined with high-bioactive particulate biocomposites. Specifically, incorporating carboxylic groups can decrease water absorption on particle surfaces and prevent the release of ions that catalyze degradation reactions under process conditions.^17−20^ However, the literature indicates that the main difficulty in functionalizing plasma with these agents lies in the stability of the treated material in aqueous environments, such as in implant conditions.^27^ When carboxylic groups are deposited under inadequate operating conditions, the rate of incorporation of functional groups is reduced, and the medium is also acidified. This acidification is of particular concern in biomedical applications due to its adverse effects on cell viability and proliferation.^27,28^ Therefore, the central question is how the excess of functionalizing agents affects the rheological, thermal, wettability, and biological properties of the biocomposite with plasma-functionalized fillers. Plasma functionalization is a nonequilibrium reaction that may not entirely consume all the initially added reagents, resulting in an excess of these substances after the process. Additionally, it is essential to control the byproducts that promote acidity in the medium to develop biomaterials suitable for biomedical applications.

Given these considerations, this study aims to provide experimental evidence on the influence of residual l-lactic acid (LA), resulting from the plasma surface modification process in the absence of a washing step, on the properties of PLA/ZnO biocomposites and its impact on the material’s applications. For this purpose, two types of PLA/ZnO biocomposites with plasma-functionalized fillers were produced: one with an excess of LA in the formulation, present in a form not chemically bound to ZnO, and one without an excess. Both formulations and the control samples (PLA and nonfunctionalized PLA/ZnO) were characterized to compare the biocomposites’ rheological, thermal, and wettability properties. Additionally, biological analyses were conducted to assess the cytotoxicity and cell proliferation of the biocomposites with and without excess, considering their potential application in biomedical contexts.

## Experimental Section

### Materials

The Poly(l-lactic acid) (PLA, Biopolymer, 2003D) used in this study was supplied by NatureWorks (Ingeo) According to the manufacturer’s specifications, it has a density of 1.24 g cm^–3^, a glass transition temperature (*T*_g_) between 55 and 60 °C, and a melting temperature (*T*_f_) of between 145 and 160 °C.^29^ Additionally, gel permeation chromatography (GPC) analysis performed by Backes et al. reported a number-average molecular weight (*M*_n_) of 118,600 g/mol and a weight-average molecular weight (*M*_w_) of 168,000 g/mol for PLA of the same grade (2003D) and manufacturer.^17^

The zinc oxide (ZnO) used as the bioceramic filler was supplied by Interprise with a purity of 99% and a average particle size of 44 μm. l-lactic acid (LA) with 85% purity (Aldrich Chemistry) was used as the functionalizing agent for ZnO surface modification through plasma treatment.

### Methods

**Figure 1:**
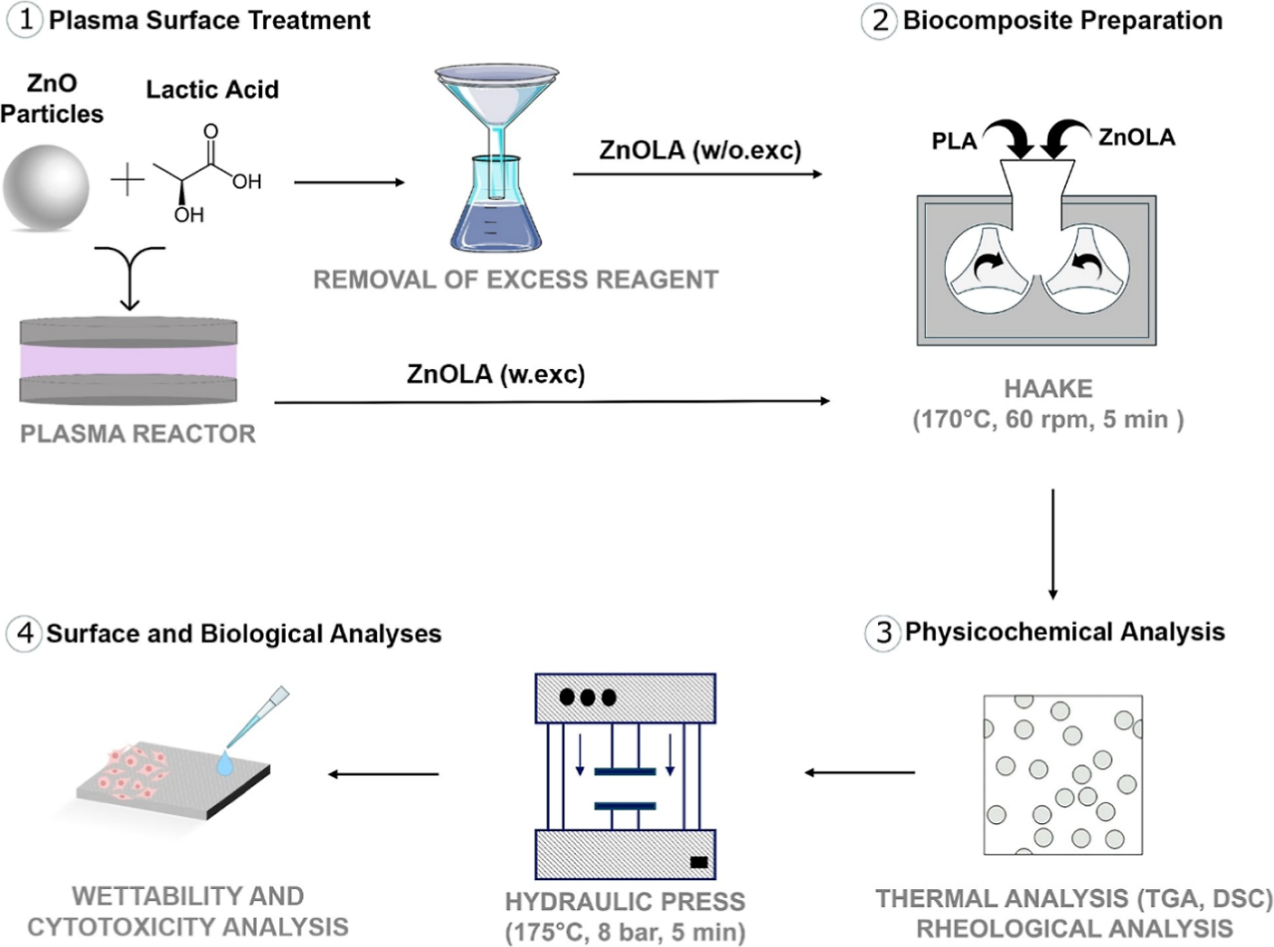
Schematic representation of the experimental procedure: (1) Plasma treatment of ZnO with l-lactic acid (LA), followed by washing (for samples without excess LA). (2) Incorporation into the PLA matrix. (3) Physicochemical analysis. (4) Surface and biological analyses of biocomposites.

The experimental procedure involved a multistep process to prepare and characterize PLA/ZnO biocomposites (Figure 1). To mitigate the catalytic degradation of PLA induced by ZnO during melt processing, the ZnO particles were plasma-treated using LA as a functionalizing agent. To evaluate the effect of excess LA on the biocomposites’ properties, the samples were divided into two groups: one was washed to remove excess LA, while the other remained unwashed . The treated ZnO particles were subsequently incorporated into a PLA matrix via melt mixing. The resulting biocomposites underwent further processing, including hot pressing to obtain the final samples. A comprehensive characterization encompassed physicochemical analyses, as well as biological evaluations such as wettability and cytotoxicity assays.

### Plasma Surface Treatment

A solid mixture of zinc oxide and l-lactic acid (ZnOLA) in a 30/70 mass proportion was manually mixed in a beaker using a spatula until no visual segregation was observed. This mixture was then dried at room temperature (RT) for 24 h. To achieve optimal homogeneity, ZnO was incrementally incorporated into LA. The resulting mixture exhibited consistency resembling a relatively thick paste. After drying at room temperature (RT), the mixture was ground using a mortar and pestle for 2 min to ensure a finer particle size and enhance material homogeneity. Then, the mixture was inserted into a homemade capacitively coupled plasma (CCP) reactor to functionalize the zinc oxide. At a base pressure of 2 × 10^–1^ Torr, argon gas was introduced at a flow rate of 36.5 sccm, reaching a working pressure of 9 × 10^–1^ Torr, measured by a Pirani gauge coupled to the reactor. The argon flow was maintained for 15 min to purge the chamber before starting the plasma discharge. Then, the RF plasma power of 35 W was chosen based on previous work, and the treatment was carried out for 5 min.^23^

After functionalization, the plasma-treated sample was divided into two groups. One group was subjected to vacuum filtration to remove excess LA from the particles. This group was then stirred in distilled water for 12 h to remove residual LA further, followed by additional vacuum filtration and washing with distilled water. The washed samples were then dried at 70 °C for 1 h before being stored in a desiccator. The second group, which retained excess functionalizing agent, was immediately placed into the desiccator after plasma treatment.

### Characterization of ZnO Filler

The ZnO was characterized by X-ray photoelectron spectroscopy (XPS) and Fourier transform infrared spectroscopy (FTIR) to confirm the functionalization of the ZnOLA particles. XPS analyses were performed using a UNI-SPECS UHV system and a Thermo Fisher Scientific (K-Alpha) spectrometer, operating at a base pressure below 5 × 10^–7^ Pa. As the excitation source, the X-ray Al K_α_ (hν = 1486.6 eV) was used with the pass energy set to 200 eV for survey and 20 eV for high-resolution scans. The inelastic noise of the high-resolution C 1s, O 1s, and Zn 2p3/2 spectra was subtracted using the Shirley method. A flood gun was employed for charge shift correction, using the C 1s hydrocarbon component (C=C/C–CH) at 284.8 eV as a reference. The composition was determined by the relative proportions of peak areas, which were corrected using Scofield atomic sensitivity factors, with an accuracy of ±5%. The spectra were deconvolved using a Voigtian-type function, with Gaussian (70%) and Lorentzian (30%) combinations. The full width at half-maximum was varied between 1.2 and 2.2 eV, and as a criterion for assigning the fitted components, a chemical shift >0.5 eV, relative to the C–CH (284.8 eV) and ZnO (1022.1 eV) references, was used.

FTIR spectra of samples in potassium bromide (KBr) tablets were obtained using a Bruker device (INVENIO-S). 32 scans were performed in the 4000–400 cm^–1^ region, with a resolution of 4 cm^–1^.

Thermogravimetric analysis (TGA) was performed using a NETZSCH STA 449C analyzer. The technique identifies the functional groups bonded to the material’s surface by analyzing mass changes as a function of temperature. The analysis was conducted at a heating rate of 10 °C/min from 25 to 1000 °C under a synthetic air flow.

#### Biocomposites Preparations

The PLA/ZnO composite formulations were processed using an internal mixer coupled to a Thermo Scientific Rheomix 600p HAAKE torque rheometer. This equipment is equipped with counter-rotating and semi-interpenetrating roller-type rotors, which homogenized the compositions at a temperature of 175 °C for 5 min at a speed of 60 rpm. Four samples were processed, distinguished by the type of surface treatment applied to the ZnO load and the post-treatment wash. Table 1 presents the samples processed along with their corresponding descriptions. After processing in the internal mixer, the samples were divided into two portions. One was subjected to thermal and rheological analysis, while the other underwent compression molding to produce test specimens. A hydraulic press model (Marconi, MA098/A) was used for compression molding, maintaining a constant temperature of 175 °C and applying a pressure of 8 bar for 5 min, then cooling until RT. The specimens obtained from this process were used in the contact angle tests and biological analyses.

**Table 1:** Sample Descriptions and Identifications

Description	Sample
Pure PLA	PLA
ZnO without LA excess	ZnOLA
PLA with 2.5% untreated ZnO	PLA + 2.5% ZnO
PLA with 2.5% ZnO with LA excess	PLA + 2.5% ZnOLA (w.exc)
PLA with 2.5% ZnO without LA excess	PLA + 2.5% ZnOLA (w/o.exc)

The processed samples were subjected to thermal, rheological, morphological analysis, wettability, and biological characterizations to assess the influence of excess functionalizing agents on the materials’ properties.

### Physicochemical Analysis

#### Thermal Analyses

TGA of the biocomposite samples was performed using a TGA 4000 analyzer from PerkinElmer. Approximately 10 mg of each sample was analyzed, with the temperature increasing from room temperature to 800 °C at a heating rate of 10 °C min^–1^, under a nitrogen atmosphere.

For the Differential Scanning Calorimetry (DSC) analysis, a TA Instruments Q2000 calorimeter was used, with a continuous nitrogen flow of 50 mL/min. The procedure consisted of two heating stages, ranging from 0 to 200 °C, at a heating rate of 10 °C min^–1^. The transition temperatures and enthalpy data of the biocomposites were determined from the results of the second heating. The degree of crystallinity was calculated during the second heating cycle using eq 1, where Δ*H*_m_ is the enthalpy of crystalline fusion, Δ*H*_c_ is the enthalpy of cold crystallization, Δ*H*_m0_ is the theoretical enthalpy of crystalline fusion for 100% crystalline PLA (93 J g^–1^),^30^ and *x* is the mass fraction of biofillers incorporated into the polymer matrix.
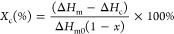
1

#### Rheological Analysis

The steady-state rheological properties of the samples were assessed through parallel plate rheometry. The objective of the analysis was to compare the viscosity levels of the materials under low shear rates (ranging from 0.01 to 100 s^–1^). For this purpose, a TA Instruments AR G2 controlled stress rheometer was used, equipped with plates with a diameter of 25 mm and a distance between the plates of 1 mm, operating under an inert atmosphere of nitrogen (N_2_) at a temperature of 165 °C.

#### Morphological Analysis

Scanning electron microscopy (SEM) was performed to evaluate the dispersion and distribution of ZnO and ZnOLA particles within the PLA matrix. The analysis utilized a Tescan Mira SEM, equipped with a Field Emission Gun (FEG), operated at an acceleration voltage of 5 kV. Initially, the samples were compressed using a hydraulic press (model MA098/A, Marconi) at 175 °C under a pressure of 7 bar for 5 min. Subsequently, the samples were cryogenically fractured along the cross-section to expose the internal structure, facilitating direct observation of the fracture surface for detailed morphological characterization.

### Surface and Biological Analyses

#### Wettability Analysis

Contact angles were measured on films prepared by pressing the samples using a hydraulic press (Marconi MA098/A) at 175 °C under 9 bar for 5 min. Wettability measurements of the films were conducted using a Biolin Scientific optical tensiometer (Attension TF3000-PLUS) with 7 μL drops of distilled water, and the angles were analyzed using OneAttension software.

#### Cytotoxicity Analysis

Preosteoblastic mouse cells MC3T3 were cultured in a medium containing 89% v/v α-MEM (Gibco) supplemented with 10% fetal bovine serum (FBS, Vitrocell) and 1% antibiotic-antimycotic (Vitrocell) in an incubator (Series II 3110, Thermo Fisher Scientific) at 37 °C, humidified and containing 5% CO_2_.

Prior to biological tests, the PLA and biocomposites were sterilized by immersing them in 70% alcohol under UV light on both sides for 15 min each; following, they were rinsed with phosphate-buffered saline (PBS) to remove the alcohol. After sterilization, the samples were placed in a 48-well plate and left in contact with 500 μL of culture medium for 24 h in an incubator at 37 °C and 5% CO_2_. After this time, the samples were transferred to a new culture plate, and 500 μL of an osteoblastic cell solution with 50,000 cells was added to each well, and the plates were incubated for 1, 7, and 14 days. The medium was changed every 2–3 days. A working solution was prepared for cell proliferation assay by diluting resazurin solution (#R7017, Millipore-Sigma) in a culture medium (1:9 dilution). After each time point, the medium was removed, 500 μL of the working solution was added to each well, and the plate was incubated at 37 °C for 4 h in the dark. Subsequently, 100 μL of the solution from all samples was transferred to a black plate where the fluorescence end point (560 nm/590 nm) was measured using a microplate reader (SpectraMax M5). Three samples of each composition were analyzed, along with three negative controls containing a cell-free resazurin solution. An autoclaved resazurin solution was used as a positive control to estimate cell viability. The results are presented in reduction percentage (%) which represents the relationship between the end point fluorescence measured in the samples (with cells) and the fluorescence of the cell-free resazurin solution (blank) and the autoclaved resazurin solution (positive control).

The cells’ characteristics on the samples’ surfaces after 14 days were assessed by scanning electron microscopy (SEM). A sample of each was removed to a new plate and washed with PBS solution. Then, 1 mL of 10% aqueous paraformaldehyde solution was added to each sample and kept for 30 min for cell fixation. Subsequently, the samples were washed with PBS and underwent a dehydration series using 50, 70, 90, and 100% ethanol concentrations. As a final step, the specimens were dried at room temperature, transferred to a stub, coated with a thin layer of gold, and analyzed using a Philips SEM model XL-30 FEG, operating at an acceleration voltage of 5 kV.

## Results and Discussion

### ZnO Functionalization

**Figure 2:**
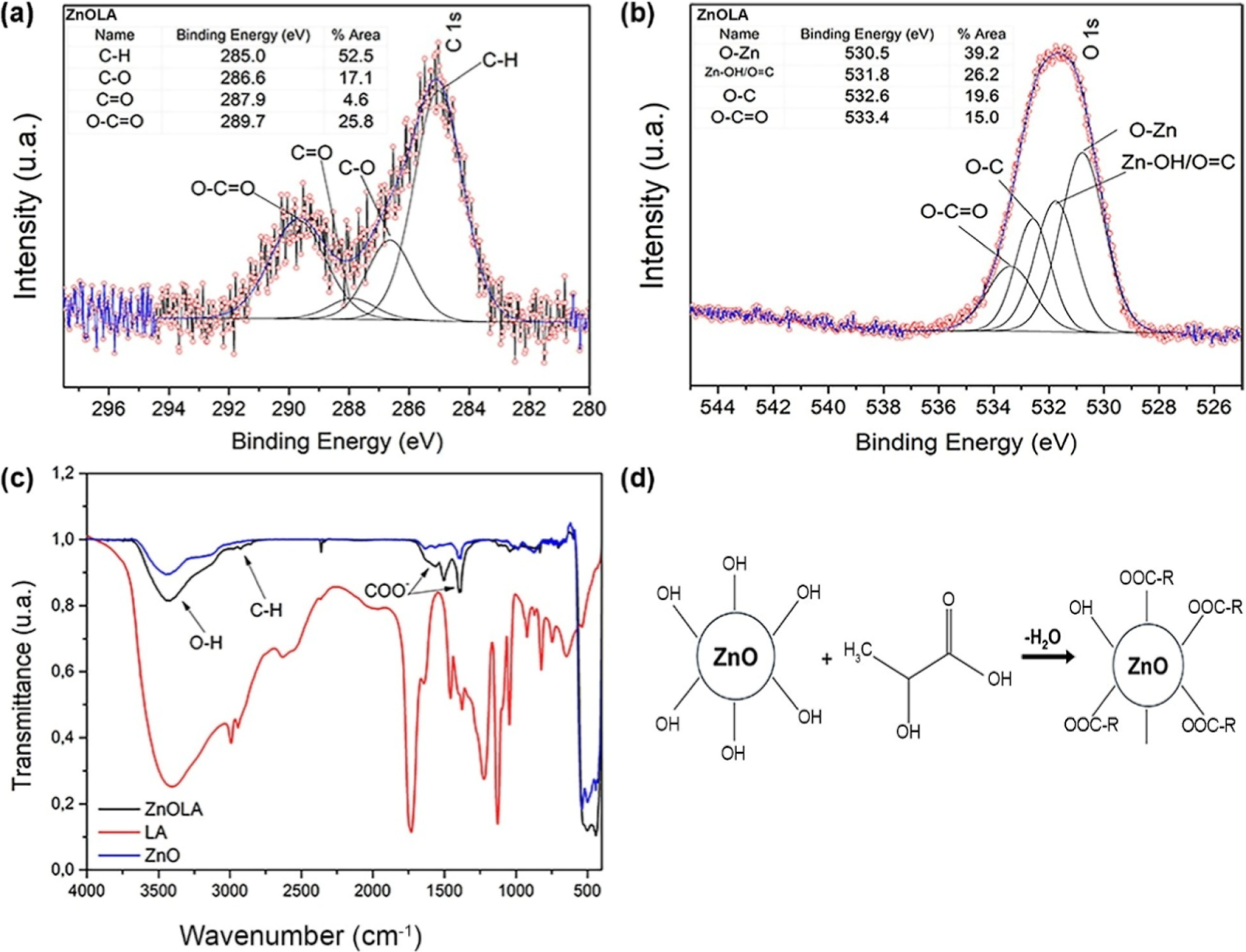
Characterization of ZnO Functionalization: (a) C 1s: 4.6% increase in carbonyl (C=O) from lactic acid (LA). (b) O 1s: increased O content (53%) and O–Zn bonds. (c) FTIR spectra. (b) Scheme of ZnO–LA preferential complexation. Figure 2d was adapted from the reference by Tang et al.^38^

XPS analyses were carried out for ZnO and the plasma-treated ZnOLA sample without excess LA to verify the effectiveness of the filler’s functionalization. Figure 2 and Table 2 summarizes the results obtained. The main components present in ZnO are the elements O 1s and Zn 2p, in addition to a contribution of the element C 1s, common in the literature, from impurities and surface contamination by hydrocarbons.^31−33^ After plasma treatment, an increase in the atomic percentage of oxygen relative to zinc was observed in the ZnOLA sample, with an O/Zn ratio of 1.8 against 1.1 for the ZnO sample. In our other work,^32^ the same behavior was observed and attributed to a sign of the particles’ functionalization. In addition, there is an increase in carbon contribution. Both changes indicate functionalization through oxygen and carbon elements in different bonds through different functional groups.^23,33^

**Table 2:** Atomic Percentage of Carbon (C 1s), Oxygen (O 1s) and Zinc (Zn 2p); and Peak Area Percentage of Functional Groups Related to C 1s and O 1s^a^

Sample	Concentration (at. %)			Peak area percentage of functional groups (area %)							
	Zn 2p	O 1s	C 1s	C–H (C 1s)	C–O (C 1s)	C=O (C 1s)	O–C=O (C 1s)	O–Zn (O 1s)	Zn–OH/O=C (O 1s)	O–C (O 1s)	O–C=O (O 1s)
ZnO	41	46	13	54.0	25.8		20.2	57.4	21.6	16.8	4.2
ZnOLA	30	53	17	52.5	17.1	4.6	25.8	39.2	26.2	19.6	15.0

a Error: ±5%.

The high-resolution C 1s spectrum for the treated sample (Figure 2a) indicates 4.6% of carbonyl groups that were nonexistent in ZnO, which comes from the functionalizing agent lactic acid. Furthermore, as the presence of oxygen atoms increases by 7 at. % in the plasma-treated sample (53 at. % against 46 at. %), the percentage of the O–Zn bond from high-resolution O 1s spectrum, as Figure 2b decreases from 57.4% (ZnO sample) to 39.2% (ZnOLA), which indicates the involvement of oxygen atoms forming bonds with other elements after treatment. In this regard, the significant increase related to functional groups from O 1s is assigned to the O–C=O group, which almost triples the original value, increasing from 4.2 to 15.0 in area percentage.

The FTIR spectra of the treated sample, shown in Figure 2c, exhibit the characteristic band of ZnO in the region below 650 cm^–1^ (Zn–O bond). C–H bonds are observed around 3000 cm^–1^, and significant peaks between 1600 and 1500 cm^–1^ and between 1400 and 1300 cm^–1^ are attributed to the asymmetric and symmetric stretching of the carboxylic acid salt functional group (COO^–^), respectively.^34^ Carboxylic acids form stable covalent bonds with metal oxide particles, mainly through carboxylate groups, and experiments indicate that the COO– group is a suitable anchor for binding to ZnO.^35^ Therefore, this result suggests that LA binding to ZnO occurs preferentially by forming surface bonds through the COOH group. The mechanism probably involved with the formation of chemical bonds between ZnO and LA is a dehydration reaction between the carboxyl group of lactic acid and hydroxyl groups on the surface of ZnO particles.^36^ The C=O bands (carboxylic groups) at 1733 cm^–1^ and C–O (elongation) between 1300 and 1000 cm^–1^, characteristic of LA, are not present, indicating both that the COOH group is in its deprotonated form and that the predominance of insertion in this plasma functionalization process is of C–H and carboxylate (COO^–^) functional groups.^36^ This result agrees with the literature, which reports that when salt comes from a carboxylic acid, it is expected that the carboxylic group bands C=O, C–O, and O–H, will be replaced by two other carbon–oxygen bands, identified as carboxylic acid salts.^37^

The displacement of the carboxylate bands in the spectra is evidence for forming metal complexes to the Zn^2+^ cation, which concludes that coordination complexes were formed.^37^ Thus, based on the results obtained by XPS and FTIR analyses, it was possible to propose a hypothesis of a preferential mechanism through which ZnO particles interact with LA. Hydroxyl groups present on the surface of particles due to the impact of moisture or water interact with the carboxylate ions coming from the functionalizing agent LA and form a complex with the Zn^2+^ ions present on the ZnO surface due to the oxygen vacancies generated in the particle by the plasma treatment.^18,38^ The functional groups are grafted onto the particle surface through several points, schematically illustrated in Figure 2d.

**Figure 3:**
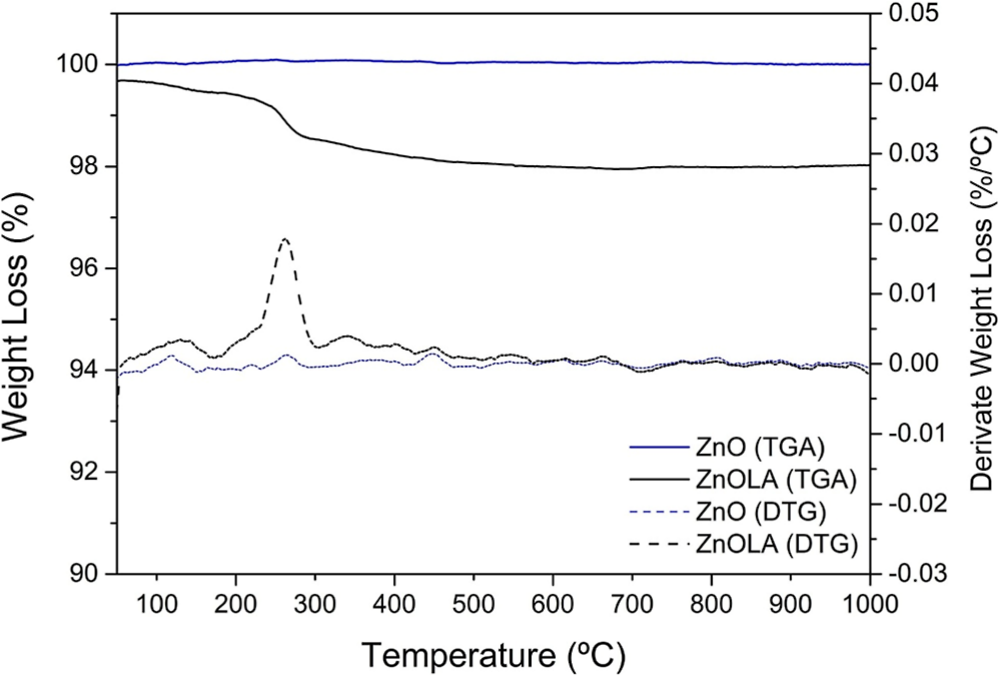
Thermogravimetry analysis (TGA) of nonfunctionalized and functionalized ZnO.

Figure 3 presents the TGA curve for both functionalized and nonfunctionalized ZnO samples. According to Barrak et al., the mass loss observed in TGA can be used to assess the quantity of functionalizing agents attached to modified particles.^33^ No mass loss was detected for the pure ZnO sample, confirming its thermal stability. In contrast, the washed ZnOLA sample (without excess) exhibited a thermal decomposition event between 200 and 300 °C, as indicated by the derivative thermogravimetry (DTG) curve. Unlu et al. reported that mass losses in metal oxides typically occur only above 500 °C.^39^ This suggests that the decomposition event observed in the washed ZnOLA sample is due to the functional groups of LA adhered to the ZnO surface. Based on this analysis, the LA content attached to the surface of the particles is estimated to be approximately 2%. Since the functionalization process was conducted with a 70:30 ZnO/LA mass ratio, the nonwashed ZnOLA sample is expected to contain approximately 30% LA. This total amount consists of two fractions: 2% of LA bound to the ZnO particles and the excess LA not removed during the washing process.

### Processing and Rheological Analysis of Biocomposites

**Figure 4:**
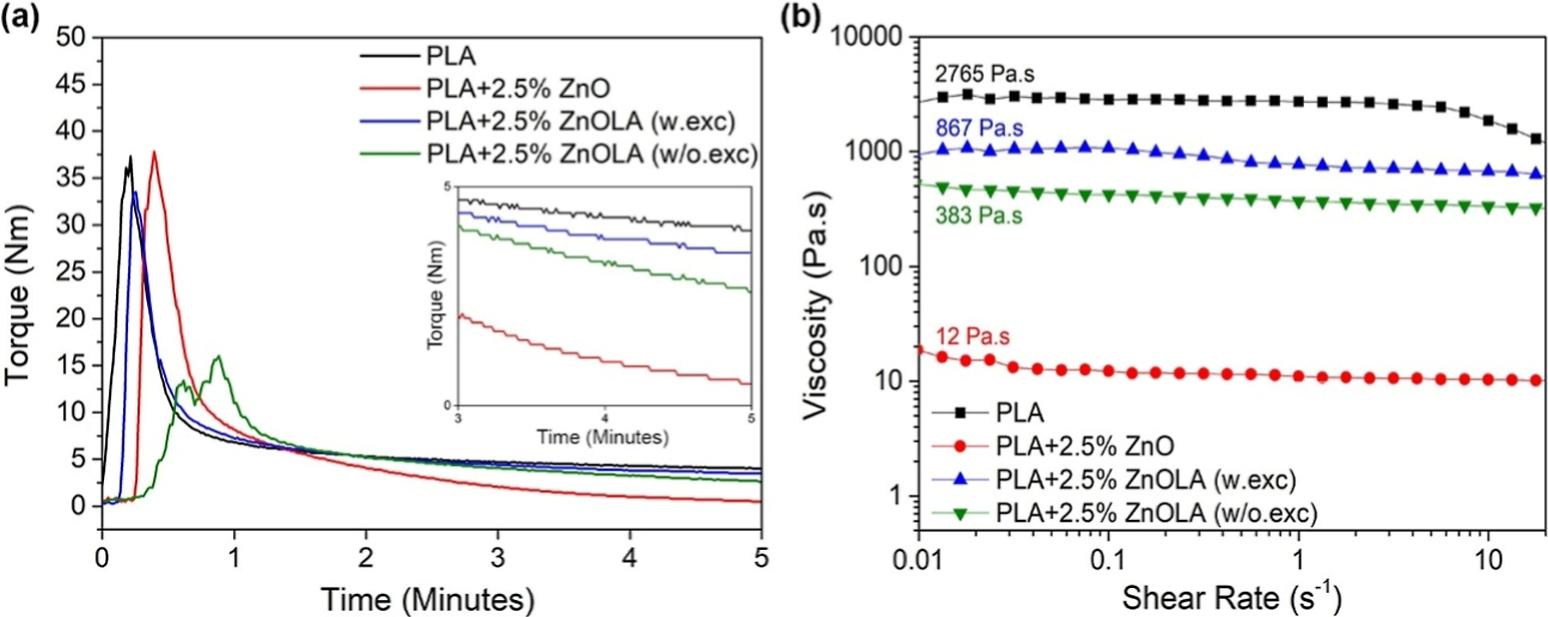
Rheological analysis: (a) torque versus time for the samples. (b) Viscosity versus shear rate from parallel plate rheometry.

Torque rheometry was utilized to process the biocomposite formulations and to provide an initial evaluation of the impact of bioceramic fillers on the rheological behavior of the PLA polymer matrix. Figure 4a illustrates torque curves (*N*_m_) versus time (minutes) for the biocomposites processed. According to the results, PLA has an equilibrium torque of approximately 4 N m after 5 min. In contrast, the non-surface-modified PLA/ZnO biocomposites (PLA + 2.5% ZnO) exhibited the lowest equilibrium torque, registering a value close to 0.5 N m at the end of the mixture process, signifying an 87.5% reduction compared to the unfilled PLA. The biocomposites containing plasma-functionalized ZnO (PLA + 2.5% ZnOLA (w/o.exc) and PLA + 2.5% ZnOLA (w.exc)) showed a less significant torque reduction than the composite with unfunctionalized ZnO.

The results confirm that incorporating plasma surface-modified ZnO allows the production of biocomposites that yield a higher torque after complete melting when contrasted with biocomposites featuring untreated filler. Quantitatively, PLA/ZnO biocomposites with plasma-functionalized filler, both with and without excess LA, showed an increase in equilibrium torque of 81% and 86%, respectively, compared to the composite without treated filler. Considering the correlation between material viscosity during processing in the internal mixer and torque on the rotors during the shear interval, the observed decrease in torque in samples with incorporated ZnO, whether surface-modified or not, suggests potential degradation in the polymer matrix during the process. Previous studies have also observed a reduction in torque values during the mixing of PLA/ZnO biocomposites. For instance, Harb et al. reported that ZnO in PLA at high processing temperatures can cause significant polymer degradation through catalyzed depolymerization, substantially decreasing thermal and mechanical properties.^15−40^ Such results highlight the need to understand how various filler treatments impact the overall material integrity during processing.

The samples’ rheological behavior was investigated using parallel plate rheometry under steady-state conditions to assess viscosity changes linked to PLA degradation. This analysis allowed for a better understanding of the influence of bioceramic fillers on the degradation process during melt processing. Figure 4b presents viscosity curves relative to a shear rate ranging from 0.01 to 100 s^–1^. All biocomposites exhibited nearly Newtonian fluid behavior between 0.01 and 10 s^–1^, with viscosity remaining constant as the shear rate increased. However, viscosity values differed among the biocomposites in this plateau region.

PLA exhibited the highest viscosity in the Newtonian regime at the low shear rate (η_0_), approximately 2765 Pa s. The composite PLA + 2.5% ZnO showed the lowest viscosity in the Newtonian plateau (12 Pa s), representing a 99% reduction compared to the polymer. Intermediate η_0_ values were observed for biocomposites with plasma-treated particles. Specifically, the viscosity for biocomposites PLA + 2.5% ZnOLA (w/o.exc) and PLA + 2.5% ZnOLA (w.exc) were 867 and 393 Pa s, respectively, indicating a reduction in η_0_ of 68% and 85% compared to PLA. These results indicate that the untreated ZnO composite samples degraded considerably. In contrast, plasma treatment, regardless of the excess functionalizing agent, resulted in lower polymer degradation. These results, observed in parallel plate rheometry, were further corroborated by the rheological behavior obtained from torque rheometry.

Shojaeiarani et al. investigated the rheological behavior of PLA/ZnO biocomposites, analyzing the complex viscosity as a function of angular frequency. They observed that as the ZnO concentration increased (from 0.5% to 1.5%), the biocomposite’s viscosity decreased in the Newtonian plateau region compared to pure PLA. The authors suggested that the higher viscosity of the sample with the lower ZnO content indicates a more efficient interaction between the nanoparticles and PLA, which could affect particle dispersion or aggregation, influencing the shear modulus. Shojaeiarani et al. also noted that at high angular frequencies (high shear rates), the viscosities of the different formulations tended to overlap, with the composite containing a higher percentage of ZnO showing a lower viscosity.^41^ In the present study, it is expected that under processing conditions, such as high shear rates, the viscosities of the biocomposites will tend to converge. However, biocomposites with plasma-treated fillers are expected to exhibit slightly higher viscosity, which could provide greater stability and control during processing. In contrast, biocomposites with untreated fillers tend to be more brittle, which may compromise their mechanical properties, highlighting the importance of functionalization for improved stability and performance of the material.

**Figure 5:**
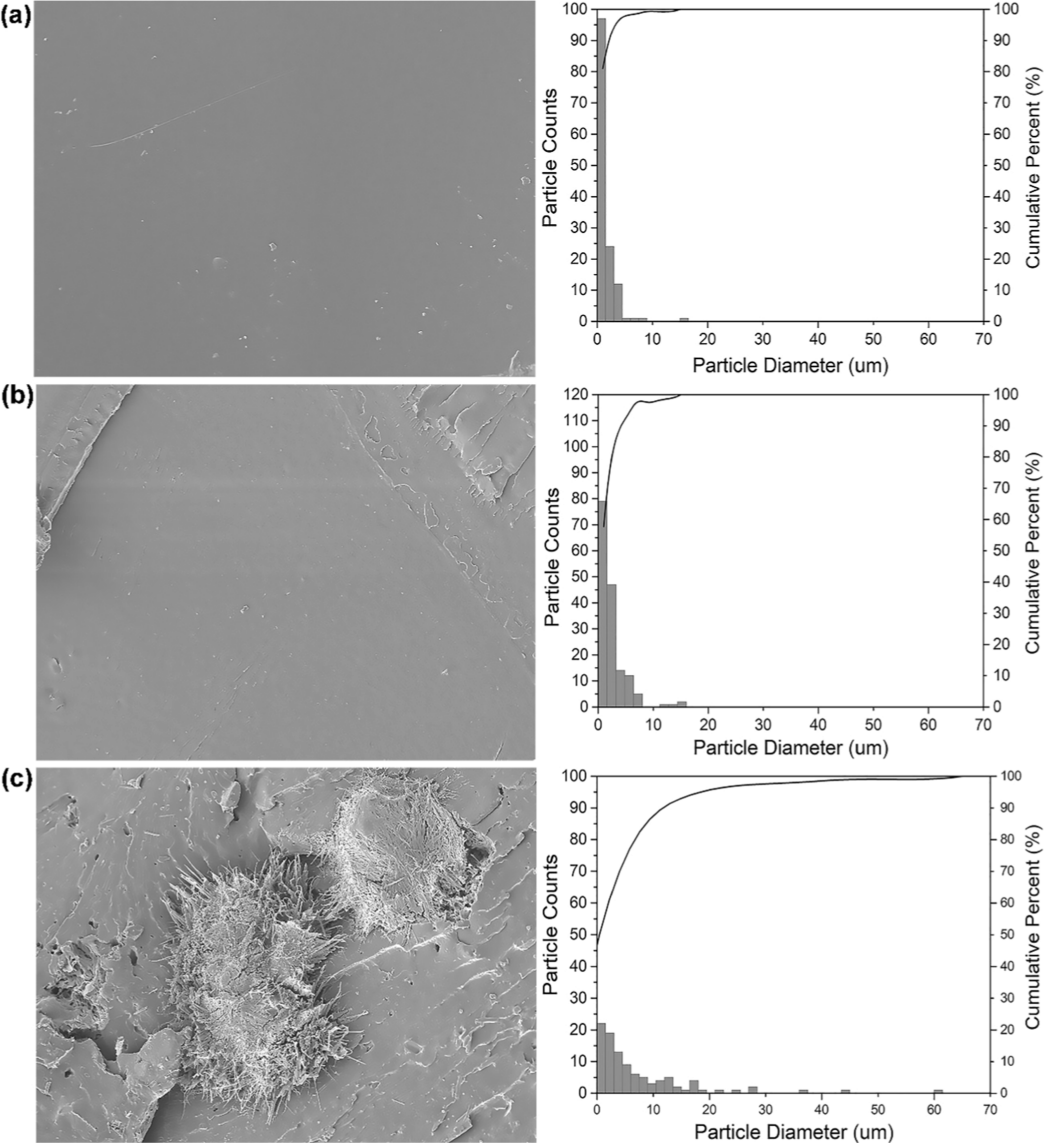
Scanning electron microscopy (SEM) micrographs and particle size distribution curves of the biocomposites: (a) PLA + 2.5% ZnO (particle size predominantly <10 μm). (b) PLA + 2.5% ZnOLA (w.exc) (agglomerates up to 60 μm). (c) PLA + 2.5% ZnOLA (w/o.exc) (particles >10 μm).

The presence of excess LA had a significant influence on the viscosity of PLA + 2.5% ZnOLA biocomposites: the composite with excess LA (PLA + ZnOLA (w.exc)) had a η_0_ of 867 Pa s. In comparison, the one without excess LA (PLA + ZnOLA (w/o.exc)) had a η_0_ of 393 Pa s, representing a reduction of approximately 55%. This phenomenon occurs because large, poorly dispersed filler agglomerates can obstruct polymer flow, increasing flow resistance. The matrix–charge interaction in biocomposites is significantly influenced by the polymer matrix’s structure and the filler surface’s characteristics, such as area, roughness, and chemical state.^42,43^ The surface treatment of the filler affects its dispersion and tendency to agglomerate, factors that primarily govern the filler–matrix interaction.^42^ In the case of the biocomposites analyzed, it was found that the excess of LA significantly influenced the poor dispersion and distribution of the particles. Figure 5 shows SEM micrographs of the surface fracture of the biocomposites analyzed, where the PLA + 2.5% ZnOLA (w.exc) sample exhibited poor particle dispersion and the presence of agglomerates. These morphological observations are consistent with the particle size distribution analysis, also shown in Figure 5. The histogram analysis revealed differences in the dispersion of ZnO particles in the PLA samples. The PLA + 2.5% ZnO sample showed the best homogeneity, with a good incorporation of the particles into the polymer matrix. In contrast, the PLA + 2.5% ZnOLA (w/o.exc) sample exhibited a slight tendency to form agglomerates, which can be observed by the higher incidence of particles with diameters greater than 10 μm. The PLA + 2.5% ZnOLA (w.exc) sample demonstrated less efficient dispersion, with larger and more agglomerated particles, showing a more pronounced shift in the distribution toward higher values, with agglomerates reaching sizes of up to 60 μm. These results suggest that the washing process to remove excess LA improves the dispersion and distribution of the particles, as it helps prevent agglomeration and enhances the overall homogeneity of the biocomposite.

#### Thermal Analyses

**Figure 6:**
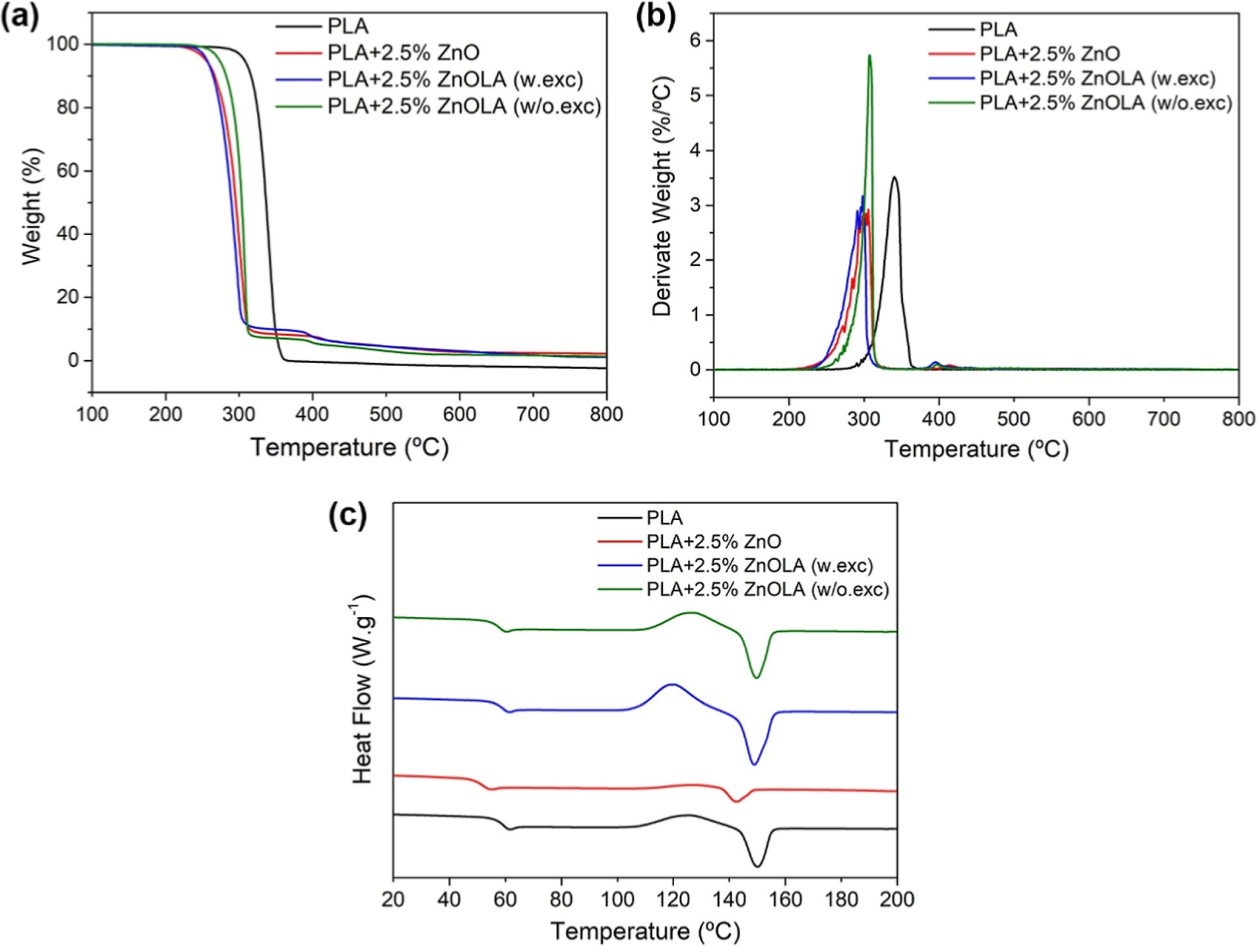
Thermal analysis of PLA and biocomposites: (a) TGA. (b) DTG. (c) DSC (curves from the second heating).

The thermal stability of the samples was evaluated by TGA under a nitrogen atmosphere, generating the curves shown in Figure 6a (TG) and Figure 6b (DTG). The PLA sample exhibited a single thermal degradation event with an onset temperature (*T*_onset_) of 312 °C and a peak temperature (*T*_peak_) of 340 °C. This is attributed to its simple chemical structure, mainly consisting of LA monomers in concordance with previously reported results.^44^ However, the decomposition temperatures (*T*_onset_ and *T*_peak_) decreased with the addition of untreated and treated ZnO, indicating the acceleration of PLA degradation with the incorporation of fillers. Comparing PLA with PLA + 2.5% ZnO, PLA + 2.5% ZnOLA (w.exc), and PLA + 2.5% ZnOLA (w/o.exc) samples revealed decreases of 46, 57, and 38 °C in *T*_onset_ temperatures, respectively. This trend of decreased thermal stability is also evident when comparing the *T*_peak_ temperatures in the DTG analysis. The reduction in thermal stability correlated with the observed weight loss attributed to the breakdown of fragile chemical bonds within the molecular chains of the composite due to the degradative processes occurring within the composite mixture.^45^

Another critical point that characterizes thermal behavior is the pattern of the TGA curve, which describes the overall degradation mechanism. In the biocomposites, weight loss occurs in two stages: the first between 250 and 270 °C and the second around 380–400 °C. In the first thermal event, the expected decomposition of the samples is evidenced by the reduction in mass as the temperature increases. In the second thermal event, there is a further decrease in the mass of the biocomposites, albeit smaller. This behavior was also observed in TGA analyses of similar formulations in previous research.^40−46^ This behavior is probably due to the decomposition and desorption of anchored groups, such as those from the functionalizing agent on the ZnO surface or the adsorption of PLA molecules on the filler.^18^

The comparison between the PLA + 2.5% ZnOLA (w/o.exc) and PLA + 2.5% ZnOLA (w.exc) reveals that the biocomposite without excess LA exhibits higher thermal stability, with a *T*_onset_ of 273 °C and *T*_peak_ of 307 °C, compared to 255 and 298 °C for PLA + 2.5% ZnOLA (w.exc), resulting in a difference of 18 °C for *T*_onset_ and 9 °C for *T*_peak_. This increased thermal stability of PLA + 2.5% ZnOLA (w/o.exc) can be attributed to the more efficient dispersion of the ZnOLA particles, which promotes better interaction between the matrix and the filler. This improvement is evident in the SEM micrographs (Figure 5), which show a more homogeneous morphology and fewer agglomerates. The literature suggests that proper particle distribution enhances the matrix and filler interaction, improving heat transfer and delaying thermal degradation.^47^ On the other hand, a higher concentration of agglomerates can create thermal stress points, acting as initial foci for degradation, thereby compromising the thermal stability of the biocomposite.^48,49^

Rheological analyses (Figure 4), particularly viscosity measurements (Figure 4b), showed that the PLA + 2.5% ZnOLA (w.exc) samples exhibited higher viscosity, which can be attributed to flow restrictions caused by the agglomeration of particles. However, this sample demonstrated lower thermal stability than PLA + 2.5% ZnOLA (w/o.exc). These findings emphasize the importance of the particle-washing process in optimizing the interaction between the matrix and the filler. Washing enhances the dispersion and homogenization of the biocomposite, thereby increasing its thermal stability, although it results in lower viscosity at low shear rates.

The enhanced thermal stability observed in PLA + 2.5% ZnOLA (w/o.exc) suggests potential advantages for biomedical applications, especially in scenarios requiring sterilization to ensure material safety. Conventional methods, such as autoclaving, which involve high temperatures (120–130 °C), are unsuitable for PLA due to its heat sensitivity and susceptibility to structural degradation.^50,51^ Alternative methods, such as gamma irradiation and ethylene oxide (EtO), are more compatible with PLA-based materials but present challenges, including polymer chain scission, molecular weight reduction, and structural alterations. The improved thermal stability in biocomposites with washed, plasma-functionalized ZnO fillers indicates increased resistance to thermally induced degradation and structural disruption, potentially mitigating the effects of sterilization processes and preserving the material’s mechanical and functional properties. These findings highlight the importance of optimizing filler functionalization to develop PLA-based biocomposites that can meet the demands of biomedical applications.

The thermal behavior of the formulations was also investigated using differential scanning calorimetry (DSC). The curves corresponding to the second heating are shown in Figure 6c. The thermal characteristics of the samples are summarized in Table 3.

**Table 3:** Thermal Characteristics of Pure PLA and PLA/ZnO Biocomposites for Second Heating.

Samples	*T*_g_ (°C)	*T*_c_ (°C)	Δ*H*_c_ (J g^–1^)	*T*_m_ (°C)	Δ*H*_m_ (J g^–1^)	*X*_c_ (%)
PLA	59.5	125	14.9	150	15.6	0.84
PLA + 2.5% ZnO	52.2	127	138	142	4.40	0.21
PLA + 2.5% ZnOLA (w.exc)	58.2	126	144	149	19.0	0.40
PLA + 2.5% ZnOLA (w/o.exc)	59.3	120	143	149	24.2	0.55

The DSC curves revealed that the PLA + 2.5% ZnO biocomposite significantly reduced the glass transition temperature (*T*_g_), indicating greater mobility of the polymer chains as a result of degradation during processing. This finding aligns with previous research,^41^ which suggested that the introduction of filler interferes with the intermolecular interactions of PLA macromolecules, probably by reducing the length of the chain, increasing chain mobility, and reducing *T*_g_. However, the formulations with plasma-functionalized ZnO (PLA + 2.5% ZnO (w.exc) and PLA + 2.5% ZnO (w/o.exc)) showed a less pronounced decrease in *T*_g_, suggesting a more efficient interaction between the ZnO particles and the polymer matrix, possibly due to the functional groups on the surface of the particles.

During the second heating, it was observed that PLA exhibited a cold crystallization temperature peak (*T*_c_), an intrinsic characteristic of this polymer due to its low crystallization rate when cooled rapidly from the molten state.^46^ In contrast, the PLA + 2.5% ZnO biocomposite did not show this phenomenon, suggesting that ZnO acts as a nucleating agent during cooling. Previous studies^52^ corroborate this observation by attributing the absence of cold crystallization to the induction of crystallization by the ZnO in the composite. Analysis of the DSC curves of the PLA + 2.5% ZnOLA (w.exc) and PLA + 2.5% ZnOLA (w/o.exc) biocomposites reveals the presence of a cold crystallization peak, indicating that the surface treatment of the filler modifies the interaction between the PLA and the ZnO particles, affecting nucleation efficiency and resulting in cold crystallization during heating. Studies such as that by Bussiere et al. investigated the effects of adding silane-treated ZnO nanoparticles to the PLA matrix, observing that these particles hindered the crystallization of the polymer by acting as antinucleating agents due to the strong interaction between the organic groups on the surface of the ZnO and the carboxylic groups of the PLA.^53^

In summary, the DSC results indicate that the PLA + 2.5% ZnOLA samples (with and without excess) showed similar thermal behavior to PLA, as evidenced by the proximity in the *T*_g_, *T*_c_, and *T*_m_ values. These findings highlight the effectiveness of the functionalization of the fillers in preserving the thermal transitions and stability of the polymer matrix, suggesting that functionalization improves the interaction between the ZnO particles and PLA, resulting in a more thermally stable material.

#### Wettability Analysis

**Figure 7:**
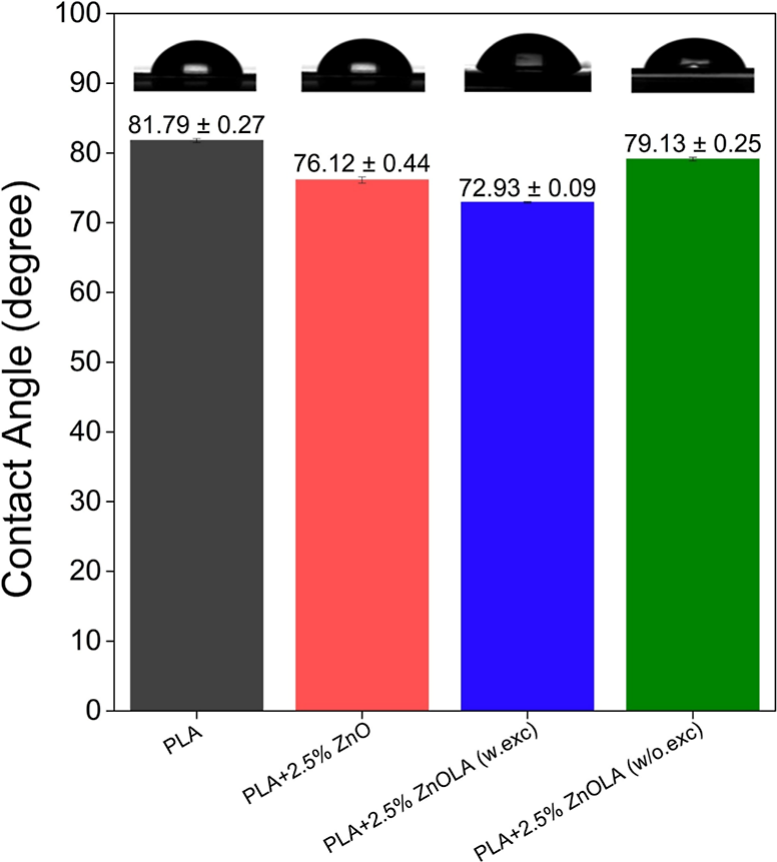
Contact angle values for PLA and PLA/ZnO biocomposites were obtained with drop deposition on the film samples.

The contact angle of the water droplets on the sample’s films was measured to investigate variations in hydrophobicity and assess the impact of excess LA on the wettability of the surfaces. As Figure 7 illustrates, PLA demonstrated hydrophilic properties, with an average contact angle of approximately 81° slightly below the 90° limit.^54^ Adding untreated ZnO to PLA reduced the contact angle to approximately 76° (76.12° ± 0.44°) for the PLA + 2.5% ZnO composite, suggesting an increase in surface hydrophilicity, probably due to ZnO’s intrinsically hydrophilic nature. This trend was most evident in the PLA + 2.5% ZnO (w.exc) sample, which recorded the lowest contact angle (72.9° ± 0.09°), possibly due to residual LA, which contains highly hydrophilic carboxyl and hydroxyl groups.^55^ The influence of washing the functionalized ZnO fillers can be seen in the approximately 6° increase in the contact angle of the PLA + 2.5% ZnO (w/o.exc) sample (79.13° ± 0.25°) compared to the PLA + 2.5% ZnO (w.exc) sample. These observations align with the findings of Guo, Xiang, and Dong (2014), who utilized citric acid to modify PLLA surfaces, reducing the contact angle from 80 to 57° and enhancing material hydrophilicity. The authors attributed this effect to the formation of hydroxyl and carboxyl polar groups and an increase in surface roughness.^56^

#### Cytotoxicity Analysis

**Figure 8:**
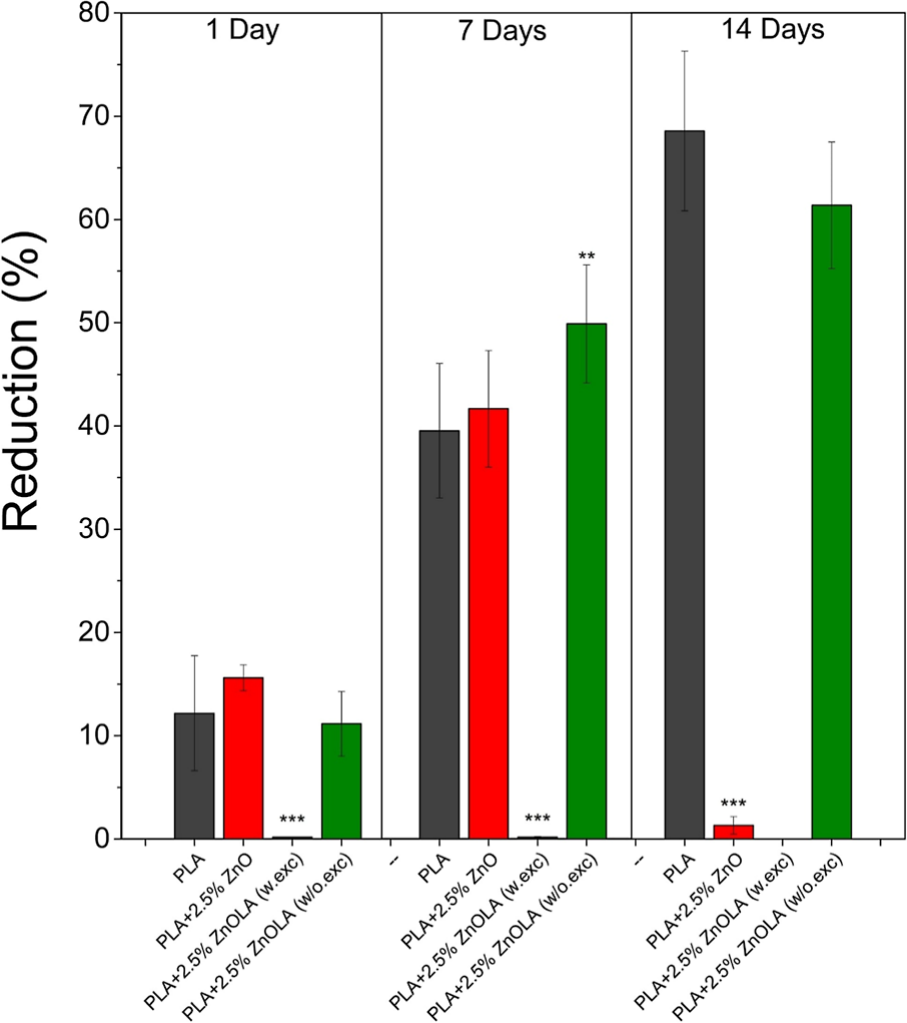
Cytotoxicity assay for PLA, PLA + 2.5% ZnO, PLA + 2.5% ZnOLA (w/o.exc), and PLA + 2.5% ZnOLA (w.exc) samples after 1 to 14 days of osteoblast culture (MC3T3-E1). Turkey’s statistical analysis was carried out about PLA: *** for *p* < 0.0001; ** for *p* < 0.01; and * for *p* < 0.05 (*n* = 6).

Cytotoxicity assay was carried out over 1 to 14 days of cell culture in the samples to investigate the effect of ZnO without treatment and with treatment (with and without excess LA); the results are illustrated in Figure 8. On the first day of testing, it was found that the PLA composite with treated filler and excess LA (PLA + 2.5% ZnOLA (w.exc)) showed no percentage increase in resazurin reduction, indicating cytotoxicity from the start of the analysis. Therefore, this was the only sample showing a significant difference from the PLA sample (*p* < 0.0001). During this same period, the other biocomposites (PLA + 2.5% ZnO and PLA + 2.5% ZnOLA (w/o.exc)) exhibited a percentage increase in resazurin reduction of 10 to 15%, showing no statistical difference from PLA. On the seventh day, the PLA, PLA + 2.5% ZnO, and PLA + 2.5% ZnOLA (w/o.exc) samples showed a substantial increase in the percentage of reduction compared to the first day, attributed to a higher number of osteoblasts metabolizing resazurin into resorufin. The highest percentage increase in resazurin occurred for the PLA + 2.5% ZnOLA (w/o.exc) composite, indicating a higher cellular response in this sample than the others. Conversely, the cellular response for the PLA + 2.5% ZnOLA (w.exc) composite remained constant after the seventh and 14th day, with no percentage increase in reduction observed throughout all assay periods. This is corroborated by the statistically significant difference observed between the percentages of resazurin reduction in PLA and the PLA + 2.5% ZnOLA (w.exc) composite (*p* < 0.0001). On the 14th day, there was an increase in the percentage of resazurin reduction for PLA and the PLA + 2.5% ZnOLA (w/o.exc) composite, indicating that the cellular response was maintained for a prolonged period of the test.

For the biocomposite with nonfunctionalized filler (PLA + ZnO), there was initially an increase in the percentage of resazurin reduction, suggesting cell proliferation in the first 7 days. However, on the 14th day of analysis, this percentage was substantially decreased.

For the central purpose of this research, the difference in the percentage of resazurin between the PLA + 2.5% ZnOLA (w/o.exc) and PLA + 2.5% ZnOLA (w.exc) samples was revealed. As observed, there was no cellular response for the PLA + 2.5% ZnOLA (w.exc) biocomposite throughout the entire assay period. In contrast, for the PLA + 2.5% ZnOLA (w/o.exc) biocomposite, an average increase of up to 60% in percentage reduction was recorded. Notably, the PLA + 2.5% ZnOLA (w/o.exc) sample demonstrated cell proliferation after 14 days of culture, suggesting that the functionalization and washing process enabled a favorable cellular environment. He et al. observed that above a concentration of 20 mmol L^–1^, LA promotes cell viability and osteogenic differentiation by distinct mechanisms, causing death mainly by acidification of the medium, i.e., pH reduction, as well as oxidative stress or interference in metabolic processes.^60^ In the present study, the excess of LA present in the PLA + 2.5% ZnOLA (w.exc) samples may have caused cell death through these mechanisms, as well as by the direct action of zinc ions in high concentration, given that the literature suggests that ZnO particles are sensitive to pH, which is evidenced by the increased release of Zn^2+^ ions at lower pH values.^61,62^ Therefore, if the zinc oxide particles are not washed after plasma functionalization, the excess LA may remain in the composition, potentially reducing the culture medium pH. This reduction in pH would increase the dissociation of Zn^2+^ ions, resulting in cell death over the incubation period.^57−59^ Trujillo et al. describe that cell proliferation is favored when the concentration of Zn^2+^ ions in the culture medium is between 25 and 50 μM, and above this concentration range, the material can be cytotoxic.^63^ These results reinforce the importance of properly controlling composite properties, including postfunctionalization washing, to ensure an adequate cellular response to tissue regeneration and repair.

**Figure 9:**
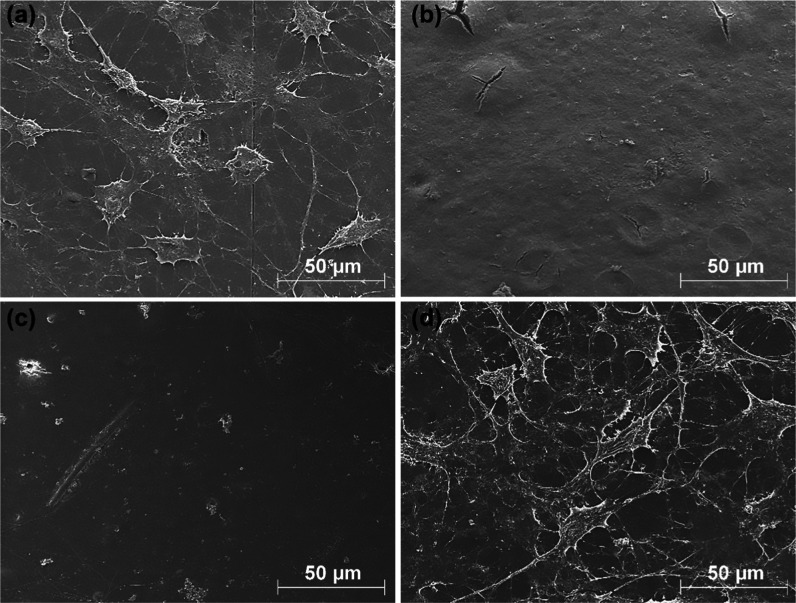
SEM images of the attachment of MC3T3-E1 osteoblastic cells to the surface of the samples: (a) PLA. (b) PLA + 2.5% ZnO. (c) PLA + 2.5% ZnOLA (w.exc). (d) PLA + 2.5% ZnOLA (w/o.exc).

Figure 9 shows the SEM images, which was used to assess the attachment of the MC3T3-E1 osteoblastic cells to the surface of the samples. The SEM images corroborate the results obtained in the cell culture test (Figure 8), showing that the osteoblastic cells are completely adhered to the surface of the PLA (Figure 9a) and PLA + 2.5% ZnOLA (w/o.exc) (Figure 9d) samples, demonstrating a high affinity for these materials. The adhered osteoblastic cells had a planar arrangement, extending evenly over the surface and showing distinct filopodia.

In contrast, a negligible number of cells were observed on the surface of PLA + 2.5% ZnO (Figure 9b) and PLA + 2.5% ZnOLA (w.exc) (Figure 9c) biocomposites. In the samples with untreated filler (PLA + 2.5% ZnO), the presence of degradation spots on almost the entire surface was noteworthy, while they were significantly absent in the other samples studied.

## Conclusions

This study investigated the functionalization of ZnO particles by plasma treatment, emphasizing the influence of excess l-lactic acid (LA) as a functionalizing agent on the thermal, rheological, wettability, and biological properties of PLA-based biocomposites. XPS and FTIR analyses confirmed the effectiveness of the functionalization, showing the formation of carboxylate groups (COO−) on the surface of the particles as a result of the interaction between the LA and the hydroxyl groups present in the ZnO. The biocomposites with plasma-treated ZnO exhibited reduced PLA degradation during processing, as evidenced by the increased equilibrium torque and the lower viscosity compared to biocomposites containing untreated ZnO. Specifically, the absence of excess LA resulted in a 55% reduction in viscosity, highlighting the significant impact of the functionalizing agent on the dispersion of ZnO particles. Furthermore, thermal analysis indicated that functionalization of ZnO, particularly after removing excess LA, helped maintain the thermal stability of the biocomposites, with a less pronounced decrease in PLA degradation temperatures. The increased viscosity observed in samples with excess LA is likely due to the restriction of chain mobility caused by the agglomeration of ZnO particles. This highlights the importance of particle dispersion quality in influencing the rheological properties and stability of PLA-based biocomposites.

However, biological analysis revealed that excess residual LA in the samples inhibited cell proliferation, underscoring the need to remove this excess for biomedical applications. This finding highlights the importance of washing the particles after functionalization, particularly when the biocomposite is intended for biological contact applications, to ensure the material’s biocompatibility. On the other hand, for applications where cell proliferation is not a key factor, washing the particles may be unnecessary, as the thermal, rheological, and wettability properties of the samples with and without excess LA remain comparable.

In conclusion, this study demonstrates that plasma functionalization of ZnO, followed by washing to remove excess LA, can mitigate PLA degradation and reduce the material’s toxicity. The introduction of functional groups onto the ZnO surface appears to aid in controlling the release of Zn^2+^ ions, which, in turn, may support cell proliferation over 14 days, as shown in cytotoxicity assays. These findings emphasize the role of the washing process in removing excess contaminants, potentially extending cell proliferation time, which is advantageous for biomedical applications requiring controlled cell growth. For other applications where biocompatibility is not a critical requirement, washing can be omitted, simplifying the preparation of the biocomposite without compromising its rheological, thermal, and miscibility properties. These results open new possibilities for utilizing PLA biocomposites in various contexts, from advanced biomedical applications to industrial uses, provided that the postfunctionalization treatment is appropriately tailored to the final application of the material. This flexibility in processing could significantly advance the practical applications of PLA-based biocomposites.
